# (Not) Being cared for in hospital settings: patients’ experience from Foucault’s perspective

**DOI:** 10.1590/0034-7167-2024-0198

**Published:** 2025-06-30

**Authors:** Débora Thais Siqueira Soares, Maria Ribeiro Lacerda, Ana Paula Hermann

**Affiliations:** I Universidade Federal do Paraná. Curitiba, Paraná, Brazil.

**Keywords:** Patient Safety, Patient Participation, Patient-Centered Care, Biopower, Patient Care, Seguridad del Paciente, Participación del Paciente, Atención Dirigida al Paciente, Biopoder, Atención al Paciente

## Abstract

**Objectives:**

to understand patients’ experience of (lack of) care in hospital settings from Foucault’s perspective.

**Methods:**

this is the partial result of research using Grounded Theory as a methodological framework, in light of Michel Foucault’s theoretical framework. Data collection was carried out with 30 participants.

**Results:**

theoretical concepts “enjoying safe care” emerged, composed of subconcepts “receiving safe care” and “feeling safe to engage in care”, and “identifying unsafe care”, with subconcepts “recognizing risks” and “feeling insecure”.

**Final Considerations:**

by amplifying patients’ voice, it was possible to capture their needs and reflect on which aspects are a reference of (in)security for them. Understanding the relations of power, knowledge and discipline in hospital settings, in light of Foucault, highlighted the complexity of interactions in care provision, favoring leading role and empowering patients for their care.

## RESUMEN

**Objetivos:**

comprender la experiencia de (falta de) atención del paciente en el ambiente hospitalario desde la perspectiva de Foucault.

**Métodos:**

este es el resultado parcial de investigación que utilizó como marco metodológico la Teoría Fundamentada, a la luz del marco teórico de Michel Foucault. La recolección de datos se realizó con 30 participantes.

**Resultados:**

surgieron los conceptos teóricos “disfrutar de un cuidado seguro”, compuestos por los subconceptos “recibir un cuidado seguro” y “sentirse seguro para cuidar”, e “identificar cuidados inseguros”, con los subconceptos “reconocer riesgos” y “sentir inseguridad”.

**Consideraciones Finales:**

ampliando la voz del paciente, fue posible captar sus necesidades y reflexionar sobre qué aspectos son un referente de (in)seguridad para él. Comprender las relaciones de poder, conocimiento y disciplina en el ambiente hospitalario, a la luz de Foucault, destacó la complejidad de las interacciones en la prestación del cuidado, favoreciendo el protagonismo y empoderando al paciente para su cuidado.

## INTRODUCTION

Hospitalization is a complex time that exposes patients to various care procedures and examinations as well as to various risk situations that predispose to the occurrence of an unexpected event^(1)^. The complexity of hospitalization is aggravated by the diversity of patients and the multiplicity of specialties and support services, which aim to treat and restore patients’ health considering their individual needs^(2)^. The more complex the care, the greater the risk of errors occurring^(3)^.

In recent years, the study of patient safety has gained prominence, especially since 2013, with the enactment of Ordinance 529/2013, which was later revoked by Consolidation Ordinance 5/2017. The topic began to be officially recognized as a concern, identifying the need to develop strategies that enable the promotion and prevention of adverse events in healthcare^(4)^.

According to the International Classification for Patient Safety (ICPS), established in 2009, risk refers to the probability of an incident occurring, which is an event or circumstance that may result in unnecessary harm to patients. In turn, patient safety is defined as reduction, to an acceptable minimum, of the risk of unnecessary harm associated with healthcare^(5)^.

The term “safe care”, analyzed from Walker’s and Avant’s perspective, refers to the promotion of healthcare provided to patients in an adequate and qualified manner, both in practical and technical and social aspects, involving the assistance and structural nature, such as the search for qualified healthcare associated with the adequacy of human and material resources^(6)^.

To achieve safe care, it is necessary to strengthen the culture of safety, invest in prevention actions, improve the health team, implement good practices, improve technologies in work settings and involve patients in their care. This improves the results for patient recovery and also significantly reduces care costs, bringing better results for healthcare service users^(7,8)^. Moreover, patients must be informed and treated as partners in their own care, as they have essential contributions to make to the safety of their care^(3)^.

To promote patient involvement in their care, it is essential to “ensure that their voice is at the center of the health movement worldwide”^(3,9)^. Thus, considering the complexity of relationships during hospitalization care, with the aim of “amplifying patients’ voice as a force for improving patient safety”^(3)^, in the search to fill gaps in current knowledge, in which care is mostly seen through institutions’ and professionals’ lens, the need arose to investigate how patients experience the safety of their care during hospitalization.

Believing that it is necessary to go beyond common knowledge, detaching oneself from the most habitual and empirical form of discourse and studying relationships more intensively^(10)^, the concepts of power relations, knowledge and discipline from Michel Foucault’s perspective were used for analysis, which provide support for research involving safe care from a more in-depth perspective^(11)^. From this perspective, knowledge and power are interdependent, with power defining what is considered knowledge and knowledge legitimizing and sustaining power, manifesting itself and controlling society through practices and discourses. In view of this, aspects of patients’ experience in the safety of their (lack of) care in hospital settings will be presented.

## OBJECTIVES

To understand patients’ experience of (lack of) care in hospital settings from Foucault’s perspective.

## METHODS

### Ethical aspects

This is a partial result of a research (doctoral thesis) conducted in accordance with national and international ethics guidelines and approved by the Research Ethics Committee, whose opinion is attached to this submission. Written informed consent was obtained from all participants involved.

### Theoretical-methodological framework

As a method reference, the Grounded Theory (GT) was used, in the Glaserian perspective, which seeks to understand social phenomena based on the data investigated, valuing the meanings and establishing a relationship between participants and the observed object^(12,13)^, based on Michel Foucault’s theoretical and philosophical framework.

### Study design

This is qualitative, interpretative research using GT. The COnsolidated criteria for REporting Qualitative research (COREQ) were considered throughout^(14)^.

### Methodological procedures

#### Study setting

Data collection was carried out at the largest public hospital in Paraná, and theoretical sampling, comprising 30 participants, was divided into three sample groups.

### Data source

The study began with the recruitment of participants for the first and second sample groups, based on a list of patients admitted in 2022, provided by the bed regulation unit at the hospital studied. The lists contained the name, age, sex, internal record and date of admission, discharge, and diagnosis of patients. After a pre-analysis, applying filters with the inclusion and exclusion criteria, the first sample group consisted of 15 patients, who spent more than 72 hours hospitalized in the referred year, with no history of reported safety incidents, aged between 18 and 59 years. Patients with cognitive disabilities, alcohol or drug use, in a situation of social vulnerability and residing outside Curitiba and the Metropolitan Region were excluded. The second sample group, made up of eight patients, follows the inclusion and exclusion criteria of the first group, and also includes those who suffered incidents with a mild degree of damage resulting from pressure injuries and falls, as these are preventable incidents that are visible to patients, allowing the development of interventions that are cost-effective.

Data analysis suggested a strong relationship between patient and healthcare professional, especially with regard to patient involvement in the safety of their own care. Thus, the third sample group was composed of seven healthcare professionals, such as physicians, physiotherapists, nurses, nursing technicians and assistants, who work in sectors where participants in the previous groups were hospitalized, even if they did not provide care to them.

#### Data collection and organization

In GT, data collection and analysis occur simultaneously^(13)^. Data collection occurred intermittently, from January 2023 to January 2024, and data organization was supported by NVivo^®^.

#### Data analysis

The data were analyzed in light of Michel Foucault’s theoretical framework, and followed the specificity and rigor of GT, in the Glaserian perspective, using substantive (open and selective) and theoretical coding, following line by line and using constant comparisons, induction-deduction, data circularity, theoretical sensitivity, formulation of hypotheses and referral to new sample groups^(15,16)^. In the case of selective coding, it results in the saturation of concepts through theoretical sampling, while theoretical codes indicate how substantive codes can relate^(17)^.

Two concepts emerged from analysis, such as “enjoying safe care” and “identifying unsafe care”, and four sub-concepts, such as “receiving safe care”, “feeling safe to engage in care”, “recognizing risks during care” and “feeling insecure while providing care”.

## RESULTS

The two concepts and their respective subconcepts will be presented in this text, the first being “enjoying safe care”, reflecting patients’ sense of security in care actions received and performed, favoring their physical and emotional recovery. Surveillance and normalization can be analyzed to understand how patients are monitored and assessed regarding their health status and compliance with care practices and how this contributes to their safety. The second concept, “identifying unsafe care”, highlights challenges that arise during the hospitalization period, such as complications, malpractice, negligence, equipment failures, lack and/or failure of communication, interpersonal conflicts, safety risks, among others.

Permeated by relationships of knowledge and power of rules, norms and routines, patients enjoy safe care and go through several challenges, which strengthen their knowledge and allow them to identify risks during the provision of care.

### Enjoying safe care

During hospitalization, patients receive a lot of care, such as hygiene, food, checking of vital signs, administration of medication and referral for tests, which are performed repeatedly. Regardless of their level of understanding, the rule, according to participants, is that they feel safe if they did not experience pain or discomfort, if there was no delay in care, if they received attention, if there was effective communication and if procedures were performed as expected or informed.

Hospitalization is surrounded by practices that normalize, discipline and monitor, but, in many cases, serve as a guide for professionals to conduct care in a safer manner.

### Receiving safe care

Dedicated attention, confirmation of data, constant monitoring, use of patients’ name when referring to them, active listening, readiness to assist, offer assistance and be present are actions carried out by professionals during patient care. These practices not only provide comfort, but also minimize patients’ fear, contributing to a more positive experience during treatment.

Participating professionals strive to ensure that information about patients’ condition, treatment plan, and relevant aspects of care are communicated in an understandable manner, helping patients identify the care received and performed correctly and safely. Furthermore, effective communication was evidenced in all three sample groups, not only promoting a positive experience during treatment, but also strengthening the relationship of trust between patients and the health team, allowing patients to feel safer and more engaged in their own care. In turn, clarity in the information shared helps patients better understand their health condition and the role of professionals in care, enabling them to make more informed decisions about their treatment and participate in their care.

### Feeling safe to engage in care

Patients’ adaptation to the new setting is facilitated by the actions of professionals who convey security during the provision of care. When these care procedures are properly communicated, an expectation is created in patients and, if they are carried out as communicated, patients feel safe, believing that they will not face risks or harm. It is common that, at some point during hospitalization, patients need to receive more intimate care from professionals, such as bathing, hygiene and even insertion of probes and catheters, which can violate very personal aspects, such as privacy. However, when this care is explained in detail, in advance, answering patients’ questions and carried out with attention and respect, although it does not completely eliminate shame or modesty, it is possible to alleviate these feelings, providing greater comfort to patients.

In addition to these aspects, demonstrating empathy and sensitivity when dealing with patients’ concerns and anxieties, being available to listen to patients’ concerns and preferences, adapting care plan as necessary, and respecting patients’ privacy during intimate care are practices of some professionals that, in participants’ perception, contribute significantly to making them feel comfortable and safe even before care is provided.

Moments of professional-patient interaction are crucial and should occur simultaneously with the care relationship and conveying of information. The safety risks to which patients may be exposed are generally explained, although, according to professionals, there is little understanding of them, and often the reinforcement of this information is not carried out ideally. In some cases, guidance only occurs after a risk has been identified or a safety incident has occurred.

A well-informed and oriented patient is aware of the care they will receive during hospitalization. It is also important to read and understand consent forms for more invasive procedures, providing patients with the resources to interact more effectively with professionals and ask questions.

The data indicate that there is a variety of perspectives among participants regarding patient involvement. Generally, decisions about treatment and care are made by healthcare professionals, who are considered to be skilled and have the necessary knowledge to determine what is best for patients at that moment. This is evidenced by patients themselves, who express confidence in the team to make decisions and choose the best care for them. Furthermore, the setting and power relations can influence how people behave and perceive themselves in the setting, and are related to the loss of individual autonomy, limiting freedom of action and thought.

### Identifying unsafe care

One of the variables that influence safe care in hospital settings is related to patients’ ability to recognize risks during care delivery. Despite patients’ different perceptions of unsafe care, there are common factors that include their involvement and that of the team, the guidance provided, the comparison between different experiences, the quality of communication and its failures as well as the discomfort experienced during the process. In this context, it is important to consider how the power/knowledge devices present in hospital settings interfere with patients’ ability to recognize unsafe care.

### Recognizing risks during care

Initially, patients may feel uncertain about perceived safety. As events repeat themselves and patients interact with other professionals and reflect on previous experiences, questions arise about the safety of certain actions.

The data revealed that there are situations in which the lack of clear information hinders care, sometimes resulting in delays, cancellations and increased hospital stays. It is common for tests or surgical procedures to be cancelled, meaning patients remain fasting for longer periods than necessary. Other occurrences include dressings being applied in different ways depending on professionals, poorly positioned drains, patient mix-ups due to lack of identification checks, unexplained tests being performed, repeated venipunctures and blood collections.

Patients identified risks of infection due to the lack of cleaning of the setting and/or hand hygiene by professionals, in addition to incidents such as falls and skin injuries. Such situations give patients a feeling of insecurity, often generated by the professionals themselves.

Regarding the subjectivity in identifying unsafe care, professionals seek to adopt safe practices in order to ensure a more positive and reliable care experience. On the other hand, patients support, stating that risks exist and, for the most part, are not properly reported.

### Feeling insecure while providing care

Identifying risks creates a feeling of insecurity in patients. Thus, adapting to the new setting requires them to change their routine. Although they understand that it is temporary, they will have to live with new people and wonder what benefits this will bring to their health.

Anxiety resulting from a lack of information or adequate physical structure suggests to patients that this may not be the best place for them to be. The limited contact with the medical team during hospitalization, which only occurs during the physician’s “visit” early in the morning, makes them feel uncomfortable. Some reports indicate that if they were asleep, they were not woken up and missed the opportunity to talk to the physician, which sometimes led to them not trusting the care of certain professionals.

Hospitalization demands a lot psychologically from patients, regardless of its duration: the unfamiliar setting leaves patients vulnerable and sensitive; added to the pain, the discomfort of being cared for by strangers, of losing privacy, becoming dependent, as well as the delay in being seen or receiving answers, can generate anxiety; discovering new problems after test results and witnessing the worsening or death of other patients result in fear, frustration, embarrassment, worry, nervousness, sadness and discomfort, and patients may feel helpless and unable to continue their treatment.

Dealing with insecurity is challenging, because although patients do not choose to be admitted to this or that institution, a mutual relationship of trust between patient and professional is necessary for them to successfully recover their health.

The identification of risks and the feeling of insecurity caused by certain actions or behaviors lead patients to express their dissatisfaction, and it is not uncommon for patients to be unaware of the reason why they should or should not undergo a certain examination or procedure. Other reasons for dissatisfaction include prolonged waiting times, the presence of a large number of patients in the same area, and the delay in receiving information, contributing to the impression of abandonment and forgetfulness. Ineffective communication emerges as the main villain in initial care at the institution, leading to a feeling of helplessness among patients.

Experiencing pain during hospitalization is a common experience, reflecting the gap between patients’ expectation of receiving adequate relief and the often frustrating reality. Professionals agree that a more effective approach to pain management is possible, but the reality often differs, resulting in intense anger and expressions of dissatisfaction from patients.

## DISCUSSION

When enjoying safe care, patients are placed in a setting that makes them feel safe and protected during care delivery. To achieve this, it is necessary not only to ensure that the technique, procedures and care are well executed, but also to build a therapeutic relationship with patients, involving acceptance, listening and connection, aiming at their comfort and well-being^(18)^.

To provide safe care, it is necessary to consider the whole, systems, setting, procedures, equipment used, organizational structure, regulations, standards, protocols, risks, economic issues, politics, teamwork, ethics, in addition to including multidisciplinary and integrated practices in this care that perceive individuals in a sociotechnical context, considering patients’ involvement in their care and the professionals in their working setting^(3,19)^.

When hospitalized, patients need professionals to meet their basic needs, and the loss of this autonomy makes them feel powerless, as they are unable to perform simple tasks, such as hygiene^(20)^. Analysis of the data from this research showed that satisfaction with this and other care received depends largely on the attention and empathy provided by professionals before, during and after care. This supports another study, in which patients praised the care team and reinforced that the commitment, attention and affection provided convey security and increase the team’s credibility^(21)^.

In the context of safe care, surveillance and normalization play significant roles. The structure of controlled settings resembles the panopticon, i.e., a surveillance model in which individuals are constantly observed, even without knowing when, creating a state of self-control and compliance. The structure allows multidisciplinary teams to work in a position to strategically observe patients and monitor them^(10)^.

For Foucault, disciplinary power manages the body without the use of direct physical force, but through a technology of the body present in society, aiming at the production of “docile” bodies^(22)^, regulating and controlling not only individual bodies, but also the population as a whole, even addressing issues such as birth control, public health and security^(23)^.

Obviously, some risks are inherent to care and, the more complex patients’ conditions, the greater the related changes or incidents^(24)^. Thus, it is known that patients can make significant contributions to safety in care^(25)^, and the experience during hospitalization is one of safety when they perceive safety measures, showing concern when they identify weaknesses^(21)^.

The initial moment of care, known as patient admission, marks the first contact with professionals and the new setting. During this time, patients feel lost due to the lack of information, and many complaints focus on the lack of adequate reception and care from professionals, and patients have the right to receive information about the results of their tests, even if they have not shown any changes^(26,27)^.

According to the results presented, unsafe care actions, such as failure to identify patients, hinder the administration of medications and interfere with the performance of tests, leading professionals to make mistakes; falls can cause injuries, traumas and bruises; inadequate positioning of patients in bed can cause pressure injuries, significantly increasing the risk of infection, the use of antibiotics and the length of hospital stay^(28)^.

These events should be a point of attention for professionals, mainly because they are classified as preventable incidents, i.e., “[...] an event that would not have occurred if a patient had received healthcare according to the normal standards of care indicated for that moment”^(5,29)^. It is important that the health team monitors the principles of infection prevention and patient safety^(30)^, paying attention to assessing patients in a unique and complex mmanner, identifying and meeting their needs individually, contributing to making care safe^(31)^.

Furthermore, correct patient identification ensures that the treatment or procedure will be performed on the person for whom it is intended, avoiding errors or mistakes in care and consequent losses^(32)^. An adequate physical structure, materials, supplies and the implementation of evidence-based practices are essential for providing safe care^(18)^. Likewise, the circulation of a large number of people in care settings interferes with care and does not meet patients’ expectations^(33)^, in addition to generating risks. Other factors related to patient dissatisfaction with the care received, which support the report of the participants in this research, concern changes in routine, noise, lighting and equipment^(25)^ - regarding these, participants are afraid of their failure.

A study conducted in southern Brazil in 2019 with nursing professionals identified that one of the essential aspects for the effectiveness of safe care is having well-established protocols and routines^(31)^. These aspects, among others, are important to understand how patients are experiencing their care, as in another study conducted in a private hospital in southern Brazil, which analyzed elements that can influence the experience of patients in relation to actions aimed at care safety in hospital settings, which includes “patient-professional interaction, communication, identification of safety protocols and the availability of the nursing team, are elements that influence the experience of patients”^(21)^.

Considering that safe care involves several actions to prevent harm to patients caused by healthcare processes, considered a strategic priority for modern health^(3,19)^, patient and family involvement and learning from lived experiences can encourage professionals to promote safety as a fundamental value^(18)^. In line with this, the Global Action Plan, in its latest edition (2020), prioritized four fundamental areas to be worked on: patient and family involvement; culture, leadership and governance; workforce safety; and learning systems^(3)^.

Patient and family engagement needs to be considered a comprehensive part of patient safety and a cornerstone of healthcare practice. This can be achieved by incorporating patients into all healthcare governance and organizational structures, by having them as a subject of community and national oversight and by giving them an equal seat at the table in global patient safety leadership and planning. This would allow patients’ and families’ voice and experience to have a powerful and beneficial influence on global and national policies, as well as on clinical and bedside practices, and by viewing all strategies through patients’ lens^(3)^.

Within the hospital, this involvement is incipient, and it is common for patients to delegate the decision regarding treatment to professionals, which is sometimes not what is expected or would not be their choice if they had the correct and detailed information about the consequences or even the quality of life after care is provided^(34)^. For many professionals, patients are seen as lacking knowledge and, therefore, considered incapable of understanding their illness or their body, making them submissive to professional care^(35)^; however, they must be included in care, treated with respect and dignity, and encouraged to question and report on matters related to their safety^(3,19)^. In this conception of power^(36)^, some professionals are not open to sharing knowledge or allowing patients to have the power of decision, with the justification of gaining respect and preventing patients from taking over as regulator, using their autonomy to negotiate their care.

Authority is described as a disciplined and productive body, the suppression of which entails the nullification of its autonomy^(10)^. Thus, care dependence is a power relationship that emerges from a network of relationships characterized as two forces that influence each other and form a continuous power game experienced in the relationship and interaction of parties^(37)^. Thus, it is necessary to include, discuss and train professors, family members and healthcare professionals to reflect on the importance of patient safety and involvement of patients and their family^(38)^, in order to help prevent incidents related to healthcare. In this context, research can increase knowledge about safe care and provide support for the health team to make decisions^(39)^, and studies that include patients’ experience and their participation and satisfaction are necessary^(32)^.

### Study limitations

The COVID-19 pandemic delayed the data collection period and made it impossible to contact some participants, which was the reason why many guests refused to participate. Therefore, the interviews were conducted remotely and, although they were conducted via video, which allowed visual contact with patients, this is still a tool that is little used, especially among people who are not familiar with technology.

### Contributions to health

The research demonstrates that patients’ experience of (lack of) care in hospital settings from Foucault’s perspective is permeated by power and knowledge, revealing that patients can identify risks, but must be informed from the beginning of hospitalization about the risk to which they may be exposed. We suggest a more collaborative and participatory approach in hospital settings, in addition to the implementation of promotion strategies that involve patients in their care, such as their inclusion in decisions related to their treatment and care. Furthermore, a transparent organizational culture is necessary, which values respect, empathy in the interaction between professionals and patients and effective communication. Raising awareness among teams, training them and making them aware of the need and importance of empowerment favors safety in care.

## FINAL CONSIDERATIONS

The research allowed us to expand patients’ voice, capture their needs and reflect on which aspects are a reference of (in)security for them. The understanding of the relations of power, knowledge and discipline in hospital settings was deepened in light of Foucault’s perspective, an association rarely used in studies in health on the perspective of the phenomenon studied – the safety of care in patients’ experience. The complexity of interactions during the provision of care was also made explicit, which allowed us to reflect on the importance of more humanized approaches, favoring patients’ protagonism and empowering them to participate in their care.

Although from patients’ perspective, their involvement is minimal, especially when it comes to treatment decisions, the data showed that some healthcare professionals play a key role by guiding, educating, and encouraging patients to follow recommendations, participate in prescribed therapies, and adopt healthy habits. They also encourage patients to ask questions, express concerns, and actively participate in their care. Hence, they provide all the necessary conditions for patients to take control of their own care and, when necessary, make decisions about their treatment options.

These actions, when performed, not only make patients more responsible for their health, but also give them autonomy and promote a partnership between patient and healthcare professional, enabling the latter to take an active role in the decision-making process regarding their health. This allows patients to modify their initial expectations regarding the hospitalization process and achieve their recovery goals more quickly.

This study demonstrated that patients are able to identify risks and perceive nuances in the care and attitudes of professionals that lead them to reflect on the need to raise awareness among teams to strengthen interpersonal relationships, communication and empower them in their care. In this way, co-participation in care tends to be safer.

Ensuring a safe care experience requires professionals to follow established protocols, promote effective communication, and be attentive to patients’ needs and concerns, contributing to an atmosphere of trust and safety in hospital settings. This is because care, when it involves patients, satisfies them and provides professionals and the institution with resources to strengthen safety practices. By integrating these ethical principles with Foucault’s concepts, it is possible to build a more fair, inclusive, and patient-centered healthcare system.
