# Supplementary material for: (Not) Being cared for in hospital settings: patients’ experience from Foucault’s perspective
Source: Rev Bras Enferm. 2025 Jun 30;78(2):e20240198. doi: 10.1590/0034-7167-2024-0198 (PMC12221270; doi:10.1590/0034-7167-2024-0198)
Supplement: Translation

# Translation: Sendo (des)cuidado no âmbito hospitalar: a vivência do paciente sob o olhar de Foucault

**DOI:** 10.1590/0034-7167-2024-0198pt

**Authors:** Débora Thais Siqueira Soares, Maria Ribeiro Lacerda, Ana Paula Hermann

**Affiliations:** I Universidade Federal do Paraná. Curitiba, Paraná, Brazil.

**Keywords:** Segurança do Paciente, Participação do Paciente, Cuidado Centrado no Paciente, Biopoder, Assistência ao Paciente

## Abstract

**Objetivos:**

apreender a vivência do paciente sobre o (des)cuidado no ambiente hospitalar na perspectiva de Foucault.

**Métodos:**

trata-se do resultado parcial de pesquisa utilizando a Teoria Fundamentada nos Dados como referencial metodológico, à luz do referencial teórico de Michel Foucault. A coleta de dados foi realizada com 30 participantes.

**Resultados:**

emergiram os conceitos teóricos “desfrutando do cuidado seguro”, composto pelos subconceitos “recebendo cuidado seguro” e “sentindo-se seguro para envolver-se no cuidado”, e “identificando o cuidado inseguro”, com os subconceitos “reconhecendo riscos” e “sentindo insegurança”.

**Considerações Finais:**

ao ampliar a voz do paciente, foi possível captar suas necessidades e refletir sobre quais aspectos são referência de (in)segurança para ele. Compreender as relações de poder, saber e disciplina no ambiente hospitalar, à luz de Foucault, evidenciou a complexidade das interações na realização do cuidado, favorecendo o protagonismo e empoderando o paciente para seu cuidado.

## INTRODUÇÃO

A hospitalização é um momento complexo que expõe o paciente a diversos cuidados, procedimentos e exames, assim como a variadas situações de risco que predispõem à ocorrência de um evento não esperado^(1)^. A complexidade da hospitalização é agravada pela diversidade de pacientes e pela multiplicidade de especialidades e de serviços de apoio, que têm como objetivo tratar e recuperar a saúde do paciente considerando suas necessidades individuais^(2)^. Quanto mais complexo é o cuidado, maior é o risco de ocorrerem erros^(3)^.

Nos últimos anos, o estudo da segurança do paciente ganhou destaque, especialmente a partir de 2013, com a promulgação da Portaria nº 529/2013, que posteriormente foi revogada pela Portaria de Consolidação nº 5/2017. O tema passou a ser reconhecido oficialmente como uma preocupação, identificando a necessidade de desenvolver estratégias que possibilitem a promoção e a prevenção de eventos adversos na atenção à saúde^(4)^.

Segundo a Classificação Internacional para a Segurança do Paciente (ICPS), instituída em 2009, risco diz respeito à probabilidade de um incidente ocorrer, sendo este um evento ou circunstância que pode resultar em dano desnecessário ao paciente. Por sua vez, segurança do paciente é definida como a redução, a um mínimo aceitável, do risco de dano desnecessário associado ao cuidado de saúde^(5)^.

O termo “cuidado seguro”, analisado na perspectiva de Walker e Avant, refere-se à promoção de uma assistência à saúde prestada ao paciente de forma adequada e qualificada tanto nos aspectos práticos quanto nos aspectos técnicos e sociais, envolvendo o caráter assistencial e estrutural, como a busca por uma assistência à saúde qualificada associada à adequação de recursos humanos e materiais^(6)^.

Para alcançar um cuidado seguro, é necessário o fortalecimento da cultura de segurança, investimentos em ações de prevenção, aperfeiçoamento da equipe de saúde, implementação de boas práticas, aprimoramento de tecnologias no ambiente de trabalho e envolvimento do paciente no seu cuidado. Isso eleva os resultados para a recuperação do paciente e, ainda, reduz significativamente os gastos com assistência, trazendo melhores resultados para os usuários dos serviços de saúde^(7,8)^. Além disso, é preciso que os pacientes sejam informados e tratados como parceiros dos seus próprios cuidados, pois têm essenciais contribuições a fazer na segurança do seu cuidado^(3)^.

Para promover o envolvimento do paciente em seu cuidado, é fundamental “assegurar que sua voz esteja no centro do movimento pela saúde em todo o mundo”^(3,9)^. Assim, considerando a complexidade das relações durante o cuidado na hospitalização, com o intuito de “amplificar a voz do paciente como uma força para a melhoria da segurança do paciente”^(3)^, na busca de preencher lacunas do conhecimento atual, em que o cuidado é visto, em sua maioria, sob a lente de instituições e profissionais, surgiu, assim, a necessidade de investigar como o paciente vivencia a segurança do seu cuidado durante a hospitalização.

Ao acreditar que é necessário ir além do conhecimento comum, desprendendo-se da maneira mais habitual e empírica do discurso e estudando com maior intensidade as relações^(10)^, utilizaram-se para análise os conceitos de relações de poder, saber e disciplina sob a perspectiva de Michel Foucault, os quais fornecem subsídios para pesquisas envolvendo o cuidado seguro em uma perspectiva mais aprofundada^(11)^. Nessa perspectiva, o saber e o poder são interdependentes, com o poder definindo o que é considerado conhecimento e o saber legitimando e sustentando o poder, manifestando-se e controlando a sociedade através de práticas e discursos. Diante disso, serão apresentados aspectos da vivência do paciente na segurança do seu (des)cuidado no âmbito hospitalar.

## OBJETIVOS

Apreender a vivência do paciente sobre o (des)cuidado no ambiente hospitalar na perspectiva de Foucault.

## MÉTODOS

### Aspectos éticos

Trata-se de resultado parcial de pesquisa (tese de doutoramento) conduzida de acordo com as diretrizes de ética nacionais e internacionais e aprovada pelo Comitê de Ética em Pesquisa, cujo parecer está anexado à presente submissão. O Termo de Consentimento Livre e Esclarecido por escrito foi obtido de todos os participantes envolvidos.

### Referencial teórico-metodológico

Como referencial método, utilizou-se a Teoria Fundamentada nos Dados (TFD), na vertente glasseriana, que busca a compreensão dos fenômenos sociais embasados nos dados investigados, valorizando os significados e fazendo relação entre o participante e o objeto observado^(12,13)^, alicerçado no referencial teórico e filosófico de Michel Foucault.

### Tipo de estudo

Pesquisa de abordagem qualitativa, interpretativa, utilizando a TFD. Consideraram-se, em todo percurso, os critérios do *Consolidated criteria for REporting Qualitative research* (COREQ)^(14)^.

### Procedimentos metodológicos

#### Cenário do estudo

A coleta de dados foi realizada no maior hospital público do Paraná, e a amostragem teórica, abrangendo 30 participantes, foi dividida em três grupos amostrais.

#### Fonte de dados

O estudo iniciou-se com o recrutamento dos participantes do primeiro e segundo grupo amostral, a partir de lista dos pacientes internados no ano de 2022, fornecida pela unidade de regulação de leitos no hospital estudado. As listas continham o nome, idade, sexo, registro interno e data da internação, alta, e diagnóstico dos pacientes. Após uma pré-análise, aplicando filtros com os critérios de inclusão e exclusão, o primeiro grupo amostral foi constituído por 15 pacientes, que passaram mais de 72 horas internados no hospital no referido ano, sem histórico de incidentes de segurança notificados, com idade entre 18 e 59 anos. Excluíram-se os pacientes com deficiências cognitivas, uso de álcool ou drogas, em situação de vulnerabilidade social e residentes fora de Curitiba e Região Metropolitana. O segundo grupo amostral, formado por oito pacientes, segue os critérios de inclusão e exclusão do primeiro grupo, e ainda incluiu aqueles que sofreram incidentes com grau de dano leve decorrentes de lesão por pressão e quedas, pois são incidentes preveníveis e visíveis aos pacientes, permitindo desenvolver intervenções que sejam custo-efetivas.

A análise dos dados sugeriu uma forte relação entre paciente e profissional de saúde, especialmente no que diz respeito ao envolvimento do paciente na segurança do seu próprio cuidado. Assim, o terceiro grupo amostral foi composto por sete profissionais de saúde, tais como médicos, fisioterapeutas, enfermeiros, técnicos e auxiliares de enfermagem, que atuam nos setores em que os participantes dos grupos anteriores estiveram internados, mesmo que estes não os tenham atendido.

#### Coleta e organização dos dados

Na TFD, a coleta e a análise dos dados ocorrem de forma simultânea^(13)^. A coleta de dados ocorreu de forma intermitente, de janeiro de 2023 a janeiro de 2024, e a organização dos dados foi apoiada pelo *software NVivo* ^®^.

#### Análise dos dados

Os dados foram analisados à luz do referencial teórico de Michel Foucault, e seguiu a especificidade e o rigor da TFD, na vertente glasseriana, empregando as codificações substantiva (aberta e seletiva) e teórica, seguindo linha por linha e usando comparações constantes, indução-dedução, circularidade dos dados, sensibilidade teórica, formulação de hipóteses e encaminhamento para novos grupos amostrais^(15,16)^. No caso da codificação seletiva, ela resulta na saturação dos conceitos por meio da amostragem teórica, enquanto que os códigos teóricos indicam como os códigos substantivos podem se relacionar^(17)^.

Emergiram da análise dois conceitos, como “desfrutando do cuidado seguro” e “identificando o cuidado inseguro”, e quatro subconceitos, como, “recebendo cuidado seguro”, “sentindo-se seguro para se envolver no cuidado”, “reconhecendo riscos durante o cuidado” e “sentindo insegurança durante a realização do cuidado”.

## RESULTADOS

Os dois conceitos e seus respectivos subconceitos serão apresentados neste texto, sendo o primeiro “desfrutando do cuidado seguro”, refletindo a sensação de segurança dos pacientes nas ações de cuidado recebidas e realizadas, favorecendo sua recuperação física e emocional. A vigilância e a normalização podem ser analisadas para compreender como os pacientes são monitorados e avaliados quanto ao seu estado de saúde e conformidade com as práticas de cuidado e como isso contribui para sua segurança. O segundo conceito, “identificando o cuidado inseguro”, destaca desafios que surgem durante o período de hospitalização, como complicações, imperícia, negligência, falhas de equipamentos, falta e/ou falha de comunicação, conflitos interpessoais, riscos de segurança, entre outros.

Permeado de relações de saber e poder de regras, normas e rotinas, o paciente desfruta de cuidado seguro e perpassa diversos desafios, os quais fortalecem seu conhecimento e permitem identificar riscos durante a realização do cuidado.

### Desfrutando do cuidado seguro

O paciente, durante a hospitalização, recebe muitos cuidados, como higiene, alimentação, verificação de sinais vitais, administração de medicamentos e encaminhamento para exames, os quais são realizados de forma repetida. Independentemente do seu nível de compreensão, a regra, segundo os participantes, é que eles se sentem seguros se não experimentaram dor ou desconforto, se não houve demora no atendimento, se receberam atenção, se houve comunicação efetiva e se os procedimentos foram realizados conforme esperado ou informado.

A hospitalização é cercada de práticas que normalizam, disciplinam e vigiam, mas, em muitos casos, servem de guia para que os profissionais conduzam o cuidado de forma mais segura.

### Recebendo cuidado seguro

Atenção dedicada, confirmação de dados, acompanhamento constante, uso do nome do paciente quando se referir a ele, escuta ativa, prontidão em atender, oferecer auxílio e estar presente são ações realizadas pelos profissionais durante o cuidado ao paciente. Essas práticas não apenas proporcionam conforto, mas também minimizam o medo deles, contribuindo para uma experiência mais positiva durante o tratamento.

Os profissionais participantes se empenham em garantir que as informações sobre a condição do paciente, o plano de tratamento e aspectos relevantes do cuidado sejam comunicados de maneira compreensível, contribuindo para que os pacientes identifiquem os cuidados recebidos e realizados de forma correta e segura. Ainda, a comunicação efetiva foi evidenciada nos três grupos amostrais, não apenas promovendo uma experiência positiva durante o tratamento, mas também fortalecendo a relação de confiança entre paciente e equipe de saúde, permitindo que o paciente se sinta mais seguro e engajado em seu próprio cuidado. Por sua vez, a clareza nas informações compartilhadas ajuda o paciente a compreender melhor sua condição de saúde e o papel dos profissionais no cuidado, capacitando-o a tomar decisões mais informadas sobre seu tratamento e participar do cuidado.

### Sentindo-se seguro para se envolver no cuidado

A adaptação do paciente ao novo ambiente é facilitada por ações de profissionais que transmitem segurança durante a realização dos cuidados. Quando esses são devidamente comunicados, cria-se uma expectativa no paciente e, se são realizados conforme comunicado, o paciente sente-se seguro, acreditando que não enfrentará riscos ou danos. É comum que, em algum momento da internação, eles necessitem receber cuidados mais íntimos por parte dos profissionais, como banho, higiene e até mesmo inserção de sondas e cateteres, o que pode violar aspectos muito pessoais, como a privacidade. No entanto, quando esses cuidados são explicados detalhadamente, com antecedência, respondendo às perguntas do paciente e realizados com atenção e respeito, embora não eliminem totalmente a vergonha ou pudor, é possível atenuar esses sentimentos, proporcionando maior conforto ao paciente.

Além desses aspectos, demonstrar empatia e sensibilidade ao lidar com as preocupações e ansiedades dos pacientes, estar disponível para ouvir as preocupações e preferências do paciente, adaptando o plano de cuidados conforme necessário, e respeitar a privacidade do paciente durante os cuidados íntimos são práticas de alguns profissionais que, na percepção dos participantes, contribuem significativamente para que se sintam confortáveis e seguros antes mesmo da realização do cuidado.

Momentos de interação profissional-paciente são cruciais, devendo ocorrer simultaneamente à relação de cuidado e à transmissão de informações. Os riscos de segurança a que os pacientes possam vir a ser submetidos são, de forma geral, explicados, embora, segundo os profissionais, haja pouco entendimento deles, e muitas vezes o reforço dessas informações não é realizado da maneira ideal. Em alguns casos, a orientação só ocorre após a identificação de um risco ou a ocorrência de um incidente de segurança.

O paciente bem-informado e orientado tem conhecimento dos cuidados que receberá durante a internação, também sendo importantes a leitura e a explicação dos termos de consentimento para procedimentos mais invasivos, proporcionando aos pacientes subsídios para interagir de maneira mais eficaz com os profissionais e realizar questionamentos.

Os dados indicam que existe uma variedade de perspectivas entre os participantes em relação ao envolvimento do paciente. Geralmente, as decisões sobre o tratamento e os cuidados são tomadas pelos profissionais de saúde, que são considerados capacitados e detentores do conhecimento necessário para determinar o que é melhor para o paciente naquele momento. Isso é evidenciado pelos próprios pacientes, que expressam confiança na equipe para tomar decisões e escolher o melhor cuidado para eles. Ademais, o ambiente e as relações de poder podem influenciar a forma como as pessoas se comportam e se percebem no meio, e estão relacionados à perda da autonomia individual, limitando a liberdade de ação e pensamento.

### Identificando o cuidado inseguro

Uma das variáveis que influenciam o cuidado seguro no âmbito hospitalar está relacionada à capacidade dos pacientes de reconhecer riscos durante a realização dos cuidados. Apesar das diferentes percepções dos pacientes quanto ao cuidado inseguro, há fatores comuns que englobam o envolvimento deles e da equipe, a orientação fornecida, a comparação entre diferentes experiências, a qualidade da comunicação e suas falhas, bem como o desconforto vivenciado durante o processo. Nesse contexto, é importante considerar como os dispositivos de poder/saber presentes no ambiente hospitalar interferem na capacidade dos pacientes de reconhecer o cuidado inseguro.

### Reconhecendo riscos durante o cuidado

Inicialmente, o paciente pode se sentir em dúvida quanto à segurança percebida. À medida que os eventos se repetem e ele interage com outros profissionais e reflete sobre experiências anteriores, surgem questionamentos sobre a segurança de certas ações.

Os dados revelaram que há situações em que a ausência de informações claras prejudica o cuidado, por vezes resultando em atrasos, cancelamentos e aumentando o período de internamento, sendo comum o cancelamento de exames ou procedimentos cirúrgicos, de modo que os pacientes permanecem em jejum por períodos mais longos que o necessário. Outras ocorrências incluem curativos realizados de maneiras distintas dependendo do profissional, drenos mal posicionados, troca de pacientes por falta de conferência de identificação, realização de exames sem explicação, punções venosas e coleta de sangue repetidas.

Os pacientes identificaram riscos de infecção devido à falta de limpeza do ambiente e/ou higienização das mãos dos profissionais, além de incidentes como quedas e lesões na pele. Tais situações trazem ao paciente uma sensação de insegurança, gerada muitas vezes pelos próprios profissionais.

No que diz respeito à subjetividade na identificação do cuidado inseguro, os profissionais buscam adotar práticas seguras, a fim de garantir uma experiência de cuidado mais positiva e confiável. Por outro lado, corroboram os pacientes, afirmando que os riscos existem e, em sua maioria, não são devidamente notificados.

### Sentindo insegurança durante a realização do cuidado

A identificação de riscos gera no paciente a sensação de insegurança. Nesse sentido, sua adaptação ao novo ambiente requer dele a transformação da sua rotina. Embora entenda que é transitório, terá de conviver com novas pessoas e fica imaginando quais benefícios isso trará à sua saúde.

A ansiedade resultante da falta de informação ou de uma estrutura física adequada sugere aos pacientes que talvez esse não seja o melhor lugar para estar. Já o pouco contato com a equipe médica durante a internação, ocorrendo somente na “visita” médica no início da manhã, gera desconforto neles. Alguns relatos indicaram que, no caso de estarem dormindo, não eram acordados e perdiam a oportunidade de conversar com o médico, o que, por vezes, levava a não confiar nos cuidados de determinados profissionais.

A internação exige muito psicologicamente do paciente, independentemente da sua duração: o ambiente desconhecido deixa o paciente vulnerável e sensível; somado à dor, o incômodo de ser cuidado por estranhos, de perder a privacidade, tornar-se dependente, assim como a demora para ser atendido ou para receber respostas, pode gerar ansiedade; descobertas de novos problemas após o resultado de exames e o fato de presenciar o agravamento ou a morte de outros pacientes resultam em medo, frustação, constrangimento, preocupação, nervosismo, tristeza e incômodo, podendo o paciente sentir-se impotente e incapaz de continuar seu tratamento.

É desafiador lidar com a insegurança, pois embora o paciente não escolha se internar nesta ou naquela instituição, faz-se necessária uma relação mútua de confiança entre paciente e profissional, para que obtenham sucesso na recuperação da saúde.

A identificação de riscos e a sensação de insegurança originada por certas ações ou comportamentos levam os pacientes a expressar sua insatisfação, não sendo raro que o paciente desconheça a razão pela qual deve ou não realizar determinado exame ou procedimento. Outros motivos de insatisfação incluem a espera prolongada, a presença de um número elevado de pacientes na mesma área e a demora para receber informações, contribuindo para a impressão de abandono e esquecimento. A comunicação ineficaz emerge como a principal vilã do atendimento inicial na instituição, levando à sensação de desamparo nos pacientes.

Sentir dor durante a internação é uma experiência comum, refletindo a lacuna entre a expectativa do paciente de receber alívio adequado e a realidade, muitas vezes frustrante. Os profissionais concordam que é possível uma abordagem mais eficaz no manejo da dor, mas a realidade, muitas vezes, difere, resultando em intensa revolta e expressões de insatisfação por parte dos pacientes.

## DISCUSSÃO

Ao desfrutar de cuidado seguro, o paciente é inserido em um ambiente capaz de fazê-lo sentir-se seguro e protegido durante a realização do cuidado. Para isso, é necessário não só assegurar que a técnica, os procedimentos e o cuidado sejam bem executados, mas também construir com o paciente uma relação terapêutica, envolvendo acolhimento, escuta e conexão, visando ao seu conforto e bem-estar^(18)^.

Para execução do cuidado seguro, é preciso considerar o todo, sistemas, ambiente, procedimentos, equipamentos utilizados, estrutura organizacional, regulamentos, normas, protocolos, riscos, questões econômicas, política, trabalho em equipe, ética, além de inserir nesse cuidado práticas multiprofissionais e integradas que percebam o indivíduo em um contexto sociotécnico, considerando o envolvimento do paciente no seu cuidado e os profissionais no seu ambiente de atuação^(3,19)^.

Quando hospitalizados, os pacientes precisam dos profissionais para atender às suas necessidades básicas, e a perda dessa autonomia faz com que eles se sintam impotentes, pois deixam de realizar tarefas simples, como higiene^(20)^. A análise dos dados desta pesquisa mostrou que a satisfação com este e outros cuidados recebidos depende muito da atenção e empatia transmitida pelos profissionais antes, durante e após o cuidado. Isso corrobora outro estudo, em que os pacientes elogiaram a equipe assistencial e reforçaram que o empenho, a atenção e o afeto dispensados transmitem segurança e aumentam a credibilidade da equipe^(21)^.

No contexto do cuidado seguro, a vigilância e a normalização desempenham papéis significativos. A estrutura dos ambientes controlados assemelha-se ao panóptico, ou seja, um modelo de vigilância no qual os indivíduos são constantemente observados, mesmo sem saber quando, criando um estado de autocontrole e conformidade. A estrutura permite que as equipes multiprofissionais trabalhem posicionadas, de maneira a estrategicamente observar os pacientes e monitorá-los^(10)^.

Para Foucault, o poder disciplinar administra o corpo sem o uso de força física direta, mas por meio de uma tecnologia do corpo presente na sociedade, visando à produção de corpos “dóceis”^(22)^, regulando e controlando não apenas os corpos individuais, mas também a população como um todo, abordando, inclusive, questões como controle de natalidade, saúde pública e segurança^(23)^.

Obviamente, alguns riscos são inerentes ao cuidado e, quanto mais complexo é o estado do paciente, maiores são as alterações ou incidentes relacionados^(24)^. Ainda nesse sentido, é sabido que pacientes podem trazer contribuições significativas sobre a segurança no cuidado^(25)^, e a experiência durante a hospitalização é de segurança quando percebem medidas de segurança, mostrando-se preocupados quando identificam fragilidades^(21)^.

O momento inicial de atendimento, conhecido como admissão do paciente, marca o primeiro contato com os profissionais e o novo ambiente. Nele, os pacientes se sentem perdidos devido à escassez de informações, e muitas queixas se concentram na falta de acolhimento e recepção adequada dos profissionais, sendo direito do paciente receber informação sobre os resultados de seus exames, mesmo que não tenham apresentado nenhuma alteração^(26,27)^.

Conforme os resultados apresentados, as ações de cuidado inseguro, como a falha na identificação do paciente, prejudicam a administração de medicamentos e interferem na realização de exames, induzindo o profissional ao erro; a queda pode ocasionar lesão, traumas e escoriações; o posicionamento inadequado do paciente no leito pode causar lesão por pressão, aumentando significativamente o risco de infecção, o uso de antibióticos e o tempo de internação^(28)^.

Esses eventos devem ser ponto de atenção dos profissionais, principalmente por serem classificados como incidentes evitáveis, ou seja, “[...] evento que não teria ocorrido se o doente tivesse recebido cuidados de saúde de acordo com os padrões normais de cuidado indicados para aquele momento”^(5,29)^. É importante que a equipe de saúde faça a vigilância dos princípios de prevenção de infecção e da segurança do paciente^(30)^, atentando-se a avaliar o paciente de forma única e complexa, identificando e atendendo às suas necessidades de forma individual, contribuindo para tornar o cuidado seguro^(31)^.

Ainda, a identificação correta do paciente garante que o tratamento ou procedimento será realizado na pessoa para a qual se destina, evitando erros ou enganos no cuidado e consequentes prejuízos^(32)^. Uma estrutura física adequada, materiais, insumos e a execução de práticas baseadas em evidências são essenciais para realização do cuidado seguro^(18)^. Da mesma forma, a circulação de grande número de pessoas no ambiente de cuidado interfere no atendimento, e não atende às expectativas do paciente^(33)^, além de gerar riscos. Outros fatores relacionados à insatisfação dos pacientes quanto ao cuidado recebido, que corroboram o relato dos participantes desta pesquisa, dizem respeito à alteração da rotina, barulho, iluminação e equipamentos^(25)^ – sobre estes, há o receio do participante quanto à sua falha.

Estudo realizado na região Sul do Brasil, em 2019, com profissionais de enfermagem, identificou que um dos aspectos essenciais para a efetividade do cuidado seguro é ter protocolos e rotinas bem estabelecidos^(31)^. Esses aspectos, entre outros, são importantes para compreender como os pacientes estão vivenciando o seu cuidado, a exemplo de outro estudo realizado em hospital privado na região Sul do Brasil, que analisou elementos que podem influenciar a experiência dos pacientes em relação às ações voltadas à segurança do cuidado no ambiente hospitalar, o que inclui “a interação paciente-profissional, a comunicação, a identificação de protocolos de segurança e a disponibilidade da equipe de enfermagem, são elementos que influenciam a experiência dos pacientes”^(21)^.

Considerando que o cuidado seguro envolve diversas ações para prevenir danos aos pacientes causados pelos processos de cuidados de saúde, considerado uma prioridade estratégica para a saúde moderna^(3,19)^, o envolvimento do paciente e da família e o aprendizado das experiências vividas podem encorajar os profissionais a promover a segurança como um valor fundamental^(18)^. Em consonância, o Plano de Ação Global, em sua última edição (2020), priorizou quatro áreas fundamentais a ser trabalhadas: envolvimento do paciente e da família; cultura, liderança e governança; segurança da força de trabalho; e sistemas de aprendizagem^(3)^.

O envolvimento do paciente e da família precisa ser considerado parte integrante da segurança do paciente, sendo um pilar da prática assistencial. Isso pode ser alcançado incorporando o paciente a todas as estruturas organizacionais e de governança de assistência à saúde, tendo-o como sujeito de supervisão comunitária e nacional e dando-lhe um lugar igual à mesa na liderança e planejamento global da sua segurança. Isso permitiria que a voz e a experiência de pacientes e familiares tivessem uma influência poderosa e benéfica em políticas globais e nacionais, como também nas práticas clínicas e à beira do leito, e que todas as estratégias fossem vistas também pela lente do paciente^(3)^.

Dentro do hospital, esse envolvimento se mostra incipiente, e é comum o paciente delegar ao profissional a decisão da terapêutica, que por vezes não é a esperada ou não seria de sua escolha se tivesse a informação correta e detalhada sobre as consequências ou até mesmo a qualidade de vida após a realização do cuidado^(34)^. Para muitos profissionais, o paciente é visto como desprovido de conhecimento e, por isso, considerado incapaz de compreender a sua doença ou seu corpo, tornando-o submisso aos cuidados profissionais^(35)^; entretanto, ele deve ser inserido no cuidado, tratado com respeito e dignidade e encorajado a questionar e relatar o que se refere à sua segurança^(3,19)^. Nessa concepção de poder^(36)^, alguns profissionais não estão abertos a compartilhar conhecimento ou permitir que o paciente tenha o poder de decisão, com a justificativa de obter respeito e impedir que o paciente assuma o seu papel de regulador, usando sua autonomia para negociar seu cuidado.

A autoridade é descrita como um corpo disciplinado e produtivo, cuja supressão acarreta a anulação de sua autonomia^(10)^. Assim, a dependência do cuidado é uma relação de poder que emerge de uma rede de relações que se caracterizam como sendo de duas forças que se influenciam mutuamente e formam um contínuo jogo de poder vivenciado na relação e interação das partes^(37)^. Dessa forma, faz-se necessário incluir, discutir e treinar professores, familiares e profissionais de saúde para refletir sobre a importância da segurança do paciente e do seu envolvimento e da sua família^(38)^, de modo a ajudar a prevenir incidentes relacionados à assistência à saúde. Nesse contexto, pesquisas podem aumentar o conhecimento sobre o cuidado seguro e dar subsídios para a equipe de saúde tomar decisões^(39)^, sendo necessários estudos que tragam a experiência do paciente e sua participação e satisfação^(32)^.

### Limitações do estudo

A pandemia de COVID-19 atrasou o período de coleta de dados e impossibilitou o contato com alguns participantes, tendo sido o motivo da recusa de muitos convidados. Por isso, as entrevistas foram feitas remotamente e, embora tenham sido via vídeo, o que possibilitou contato visual com paciente, essa ainda é uma ferramenta pouco utilizada, principalmente entre as pessoas não integradas à tecnologia.

### Contribuições para a área da saúde

A pesquisa demonstra que a vivência do paciente sobre o (des)cuidado no ambiente hospitalar na perspectiva do Foucault é permeada pelo poder e saber, revelando que o paciente pode identificar riscos, mas deve ser informado desde o início da hospitalização sobre o risco aos quais pode ser submetido. Sugerimos uma abordagem mais colaborativa e participativa no ambiente hospitalar, além da implementação de estratégias de promoção que envolvam o paciente no seu cuidado, como sua inclusão nas decisões relacionadas ao seu tratamento e seu cuidado. Ainda, faz-se necessária uma cultura organizacional transparente, que valorize o respeito, a empatia na interação entre profissionais e pacientes e a comunicação eficaz. Sensibilizar as equipes, capacitando-as e conscientizando-as sobre a necessidade e importância do empoderamento favorece a segurança no cuidado.

## CONSIDERAÇÕES FINAIS

A pesquisa permitiu ampliar a voz do paciente, captar suas necessidades e refletir sobre quais aspectos são referência de (in)segurança para ele. Aprofundou-se a compreensão das relações de poder, saber e disciplina no ambiente hospitalar à luz da perspectiva de Foucault, associação pouco realizada em estudos na área da saúde sobre a perspectiva do fenômeno estudado – a segurança do cuidado na vivência do paciente. Também se explicitou a complexidade das interações durante a realização do cuidado, o que permitiu refletir sobre a importância de abordagens mais humanizadas, favorecendo o protagonismo do paciente e emponderando-o a participar de seu cuidado.

Embora, na perspectiva do paciente, seu envolvimento seja mínimo, especialmente no que diz respeito às decisões sobre o tratamento, os dados evidenciaram que alguns profissionais de saúde desempenham um papel fundamental, orientando, educando e encorajando os pacientes a seguir as recomendações, participar das terapias prescritas e adotar hábitos saudáveis. Além disso, incentivam os pacientes a fazer perguntas, expressar suas preocupações e participar ativamente do seu cuidado. Dessa forma, fornecem todas as condições necessárias para que os pacientes possam assumir o controle de seus próprios cuidados e, quando necessário, tomar decisões sobre suas opções de tratamento.

Essas ações, quando ocorrem, não apenas tornam os pacientes mais responsáveis por sua saúde, mas também lhes concedem autonomia, além de promover uma relação de parceria entre paciente e profissional de saúde, capacitando aquele a assumir um papel ativo sobre sua saúde no processo de tomada de decisão. Isso permite que os pacientes modifiquem suas expectativas iniciais em relação ao processo de internação e alcancem mais rapidamente seu objetivo de recuperação.

Este estudo demonstrou que pacientes são capazes de identificar riscos e perceber nuanças do cuidado e atitudes dos profissionais que os levam a refletir sobre a necessidade de sensibilizar as equipes para fortalecer a relação interpessoal, a comunicação e emponderá-los no seu cuidado. Dessa forma, a coparticipação do cuidado tende a ser mais segura.

Garantir uma experiência de cuidado segura implica aos profissionais seguir protocolos estabelecidos, promover a comunicação eficaz e estar atentos às necessidades e preocupações dos pacientes, contribuindo para uma atmosfera de confiança e segurança no ambiente hospitalar. Isso porque o cuidado, quando envolve o paciente, o satisfaz e fornece aos profissionais e instituição subsídios para fortalecer práticas de segurança. Ao integrar esses princípios éticos com os conceitos de Foucault, é possível construir um sistema de saúde mais justo, inclusivo e centrado no paciente.
